# A Vision-Based Driver Assistance System with Forward Collision and Overtaking Detection †

**DOI:** 10.3390/s20185139

**Published:** 2020-09-09

**Authors:** Huei-Yung Lin, Jyun-Min Dai, Lu-Ting Wu, Li-Qi Chen

**Affiliations:** 1 Department of Electrical Engineering, Advanced Institute of Manufacturing with High-Tech Innovation, National Chung Cheng University, Chiayu 621, Taiwan; 2 Department of Electrical Engineering, National Chung Cheng University, Chiayi 621, Taiwan; jyunmin@godel.ee.ccu.edu.tw (J.-M.D.); luting@godel.ee.ccu.edu.tw (L.-T.W.); liqi@godel.ee.ccu.edu.tw (L.-Q.C.)

**Keywords:** advanced driver assistance system, forward collision warning, overtaking vehicle identification, lane change detection

## Abstract

One major concern in the development of intelligent vehicles is to improve the driving safety. It is also an essential issue for future autonomous driving and intelligent transportation. In this paper, we present a vision-based system for driving assistance. A front and a rear on-board camera are adopted for visual sensing and environment perception. The purpose is to avoid potential traffic accidents due to forward collision and vehicle overtaking, and assist the drivers or self-driving cars to perform safe lane change operations. The proposed techniques consist of lane change detection, forward collision warning, and overtaking vehicle identification. A new cumulative density function (CDF)-based symmetry verification method is proposed for the detection of front vehicles. The motion cue obtained from optical flow is used for overtaking detection. It is further combined with a convolutional neural network to remove repetitive patterns for more accurate overtaking vehicle identification. Our approach is able to adapt to a variety of highway and urban scenarios under different illumination conditions. The experiments and performance evaluation carried out on real scene images have demonstrated the effectiveness of the proposed techniques.

## 1. Introduction

Nowadays, traffic safety has become a significant issue due to the rapid worldwide growth in the number of vehicles. Most vehicles move at high speed, particularly on the highways, to provide the convenient transportation and shorten the travel time. Nevertheless, this will come with the danger of potential traffic accidents due to the road users as well, and a considerable number of accidents are caused by fatigue or distraction of the drivers. Thus, the development of driver assistance systems is important and with great practical values in today’s safety trend. In general, these systems are used to improve the traffic efficiency and keep away from traffic accidents. They also serve as the key components of autonomous driving vehicles, especially for the long distance highway travel [1]. Several related fields, including intelligent vehicles, advanced safety vehicles, and intelligent transportation systems, have attracted researchers and practitioners to investigate the driver assistance technologies.

According to the report of Taiwan Area National Freeway Bureau [2], the major traffic accidents in the highways are caused by the result of “improper lane changes” and then “not paying attentions to the state in front of the vehicle”. Most of the dangerous situations are due to insufficient safe driving distances, lane changing, and vehicle overtaking. The vehicle drivers usually assess the surrounding traffic before changing lanes, and make turns after checking their rear view and side mirrors. Thus, an effective front and side monitoring with rapid detection of vehicles is important. It should be able to provide the necessary surrounding information to the driver and issue the in-time warnings to avoid possible collision.

In recent years, the advanced driver assistance systems (ADAS) have achieved great advances in vehicle safety issues. Many techniques have been proposed for better environment perception and decision making. Some typical applications for important ADAS modules include lane change detection (LCD), forward collision warning (FCW), and overtaking vehicle identification (OVI) [3,4,5]. LCD is to provide the vehicle in-lane position or lane departure information by the identification of road markings. The objective of FCW is to perform front object detection and generate an alert to the driver to prevent the impending collision. On the other hand, OVI aims to detect the vehicles approaching from the rear, and provides an in-time warning for making a turn or changing lanes. At the present, many high-end vehicles are equipped with ADAS directly from car manufacturers, while there also exist after-market onboard traffic recorders equipped with various ADAS functions.

For the development of advanced driver assistance systems, many different sensors such as radar, lidar, and a camera, are adopted. Laser and radar are two commonly used sensors for distance measurement and forward collision warning. There are many advantages including the capability of detecting long range obstacles, even under rainy/cloudy weather conditions or at night. However, in addition to the expensive hardware cost, these approaches also lack of sufficient object recognition ability to analyze the traffic scenes. To identify individual lanes and drivable areas, visual sensors are commonly adopted for road surface marking detection. The transportation infrastructure can also be integrated with GPS-based vehicle localization for lane change assistance. For blind spot or rear obstacle detection, radar-based sensing techniques are commonly used [6].

To reduce the cost of driver assistance systems, vision-based techniques which adopt low-cost cameras as sensing devices have been proposed in recent years [7]. They have attracted considerable attentions due to not only the inexpensive hardware compared to radar or lidar technologies, but also the significant advances and maturity in computer vision and machine learning algorithms. The rich sensing information of the environment provided by the cameras can also be used to extend the functions of driver assistance systems. Some applications under investigation include traffic light detection [8], traffic sign recognition [9], vehicle identification [10], vehicle speed detection [11], pedestrian recognition [12], overtaking detection [13], and parking assistance [14], etc. Thus, despite the depth measurement precision under varying illumination conditions, it still has great potential to use image-based approaches for the vehicular system development.

**Figure 1:**
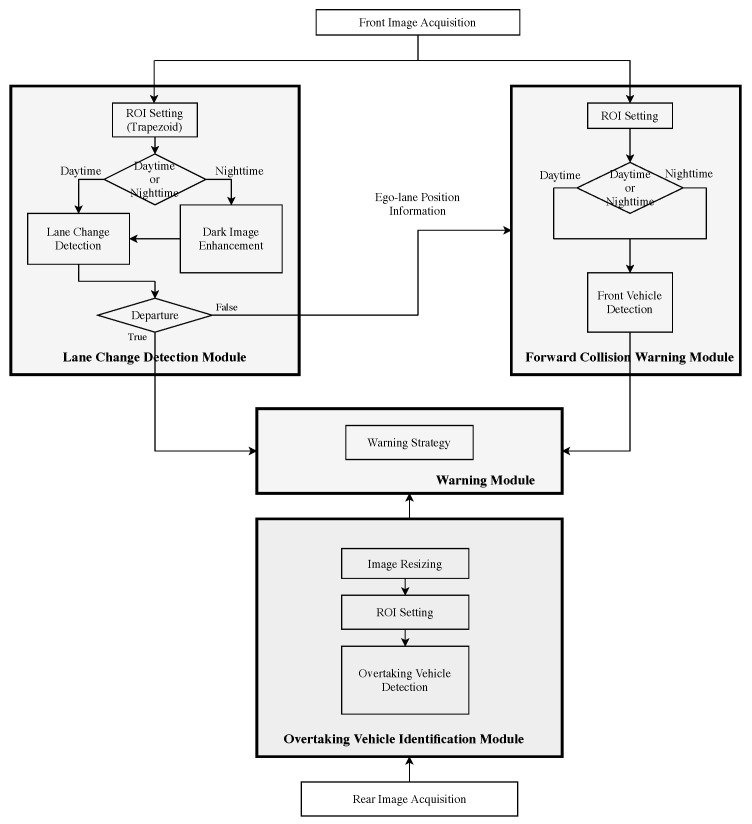
The schematic diagram of the proposed vision-based driver assistance system. It consists of three modules: lane change detection, forward collision warning, and overtaking vehicle identification. Daytime and nighttime driving scenarios are also considered in the implementation.

The challenges of lane marking and vehicle detection include complex road conditions, dynamic scenes, and changing illumination. Moreover, the robustness and real-time are also the key indicators of the algorithm design and system implementation. Thus, we mainly adopt the feature-based approaches in this work, with the learning-based methods for validation purposes. A schematic diagram of the proposed system is depicted in Figure 1. The lane markings and front vehicles are detected and identified first. New algorithms are proposed to improve the detection accuracy for complex scenarios. A symmetry analysis technique is then adopted for the verification of vehicle detection results. For overtaking vehicle detection, a motion-based method is used for continuously tracking [15], followed by a convolutional neural network (CNN) for target region classification and identification.

In this paper, we propose an advanced driver assistance system using two cameras to capture image data from the front and rear views of the vehicle. Our objective was to develop efficient and robust techniques for lane change detection, forward collision warning, and overtaking vehicle identification. In the past few decades, the computation of image data has been greatly reduced due to the prevalence of graphics processor unit. The hardware progress makes the visual data processing approaches suitable for ADAS applications. Unlike the conventional radar-based approaches [16], our vision-based system can enhance the performance of existing object identification and lane detection techniques. When the camera is properly mounted and calibrated, it can be further integrated with other computer vision algorithms to form better safety protections to the drivers without extra hardware cost. The main contributions of this work include the following.

- A driver assistance system including lane change detection, forward collision warning, and overtaking vehicle identification is presented.
- We propose a new method for front vehicle detection using an adaptive ROI and the CDF-based verification.
- The proposed overtaking detection approach with CNN-based classification is used to solve the difficult repetitive pattern problem.
- The vision-based system is developed with comprehensive camera calibration procedures and evaluated on real traffic scene experiments.

## 2. Related Work

In recent years, many vision-based techniques for advanced driver assistance systems are investigated. The versatility of vision systems provides rich information such as color, shape, and depth information at a low hardware cost. The previous work on lane change detection can be categorized into three different approaches: the feature-based, model-based, and inverse perspective transform techniques. In feature-based methods, the road markings [17,18] or the lane lines [19] are taken as low level features to locate the lane position in the image. On the other hand, pre-defined curves (e.g., straight line or parabola functions [20], etc.) are used to approximate the lanes via parameter estimation in model-based methods. The inverse perspective transform methods take the 3D space as input and perform a linear transformation to a 2D space for processing [21,22]. In addition to these approaches, there are other techniques, such as transforming front-view images to top-view images for straight line detection [23] and lane detection and tracking using deformable models [24].

The existing techniques for forward collision warning include feature-based [25], stereo-based [4,26], motion-based [27,28], and learning-based [29,30] approaches. To detect the front vehicle, the underneath shadow is commonly adopted as a pattern from visual cues [31]. The shadow image region obtained from thresholding or gradients can be used to locate the front vehicle. Since the shadow intensity depends on the illumination condition of the scene, a fixed threshold is in general not suitable for detection. Adaptive thresholds will be required for different environments. Moreover, the candidate vehicle region associated with the shadow needs to be verified by symmetry [32,33] or training data. In [34], the feature matching and 3D feature clustering are used to select reliable features for a stereo-based technique. A transform model is estimated using modified RANSAC for vehicle detection and tracking. Lai et al. use the characteristics of regional color change and optical flow to identify the vehicle for collision avoidance [35]. Chen et al. adopt a multi-layer artificial neural network for robust detection of critical motion from vehicle contextual information [36]. Although the learning-based approach provides better results, it usually has high computational complexity and hardware requirements.

For overtaking detection, the cameras can be installed in the front, rear, or sides of the vehicle. Chen et al. and Hultqvistet et al. propose efficiency vehicle identification algorithms using optical flow [37,38]. A single camera is placed in the front of the vehicle, and optical flow is evaluated along the lines parallel to the motion of the overtaking vehicles. Based on the features tracked along the lines, the position of the overtaking vehicle is estimated. However, due to the limited field-of-view of the camera mounted in the front, these methods are not able to notify the driver about the occurrence of overtaking in advance. Alternatively, Alonso et al. present an overtaking detection technique for the blind-zone using the cameras mounted on the side-view mirrors [39]. Wu et al. also utilize the cameras installed below the side-view mirrors to develop an embedded blind-zone security system [40]. The low-level features such as shadows, edges, and headlights are first detected for vehicle localization, and then followed by tracking and behavior analysis.

## 3. Lane Change Detection

In our lane change detection approach, the input takes about a half of the original image with the unnecessary region such as the sky and buildings removed. The region of interest (ROI) is further defined before performing the low-level feature-based lane marking detection algorithm. To choose a suitable ROI for line detection, most current methods use a range above the free space and under the horizon. However, this can easily cover the extra regions in both sides of the image, which are usually not necessary for processing. To deal with this problem, a heuristic trapezoid region is set as our ROI for line detection.

### 3.1. Lane Marking Detection in Daytime

For a given ROI, a gradient map is obtained with the Sobel operator, and used to detect the lane markings and vehicle shadows for lane departure and forward collision warning. The gradient direction of each pixel is also used to restrict the orientation of the lines. To identify the road lanes, the gradient image is binarized for line detection. The connected component labeling is then carried out to reduce noise in the binary image. Due to the different characteristics of the scenes associated with different distances, the image is partitioned to upper, middle, and lower regions. The associated edge image regions are derived with different thresholds. Line detection using Hough transform is then carried out with the orientation constraints specified by the vanishing point to identify the lane markings.

**Figure 2:**
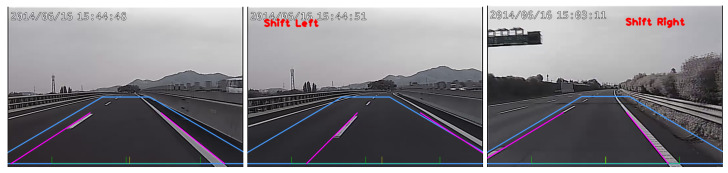
The illustration of lane departure status. **Left**: normal. **Middle**: shift left. **Right**: shift right. In the images, a trapezoid region is set as the region of interest (ROI) for line detection.

In this work, we restrict the interested gradient directions in 15°–85° and 95°–165° for the left and right lane markings, respectively. This range can be used to suppress the horizontal and vertical lines frequently appeared in the environments with man-made structures. Figure 2 shows the lane change status, *left*, *right*, and *normal*, based on the Hough line detection results. For the real scene applications, the lane shift direction cannot be uniquely determined without sequential observation. To increase the robustness of lane change detection, a finite state machine is adopted. In our approach, only the ‘normal’ state can change to ‘shift right’ or ‘shift left’, and update the lane change status. As shown in the experiments, this method can successfully reduce the false alarms due to the lane markings occasionally occluded by the front vehicles.

### 3.2. Image Enhancement for Low Light Scenarios

To increase the contrast of an image, the gamma correction on the intensity is commonly adopted. In this work, a variant of gamma correction function is proposed to solve the contrast problems associated with low light traffic scenarios. The conventional gamma correction function utilizes a nonlinear operation to encode and decode luminance or tristimulus values in video or still image systems. It is defined by the power-law conversion

(1) $$I_{out}=c\cdot I_{in}^{\gamma }$$

where the input ($I_{in}$) and output ($I_{out}$) are non-negative values and *c* is a constant.

**Figure 3:**
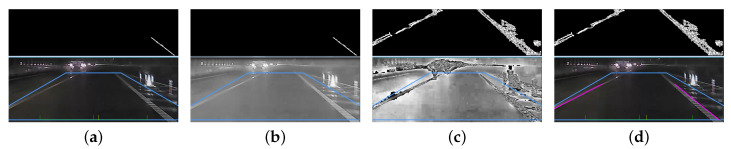
The comparison of conventional gamma correction and proposed gamma correction results. (**a**) The detection result with no gamma correction; (**b**) the detection result using conventional gamma correction; (**c**) the input image using the proposed gamma correction; (**d**) the result using the proposed gamma correction.

For the regular images with the intensity range of 0 to 255, the constant *c* is given by $c=255/255^{\gamma }$, where γ is less than 1. This process adjusts the pixel value and makes it brighter than its original intensity. We use the gamma correction function defined by

(2) $$I_{out}=\left\{\begin{array}{cc} I_{in}\cdot I_{in}^{\gamma }, & \mathrm{if}\;I_{in}\leq T\\ (\left\lfloor I_{in}\cdot I_{in}^{\gamma }\right\rfloor )-256, & \mathrm{otherwise}. \end{array}\right.$$

to calculate the value of each pixel. In the above equation, the constant *c* is changed to $I_{in}$ for lane marking detection specifically in low light conditions. The threshold and gamma value are set as T=52 and γ=0.4 empirically for general night scenes. Figure 3 shows the results using different gamma correction approaches. The lane marking detection result using the proposed gamma correction formulation is shown in Figure 3d.

## 4. Forward Collision Warning

**Figure 4:**
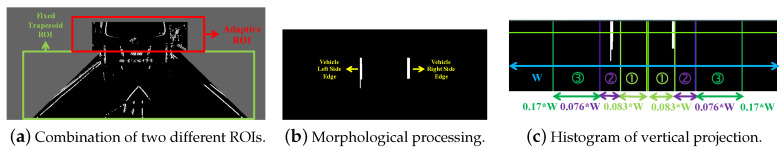
The front vehicle detection using a vertical edge pair. A combination of an adaptive ROI and a fixed trapezoid is used.

In our forward collision warning module, the input is the gradient image and lane marking detection results. The cast shadow is used for front vehicle detection since it is the most prominent feature in daytime. We propose the combination of an adaptive ROI and a fixed trapezoid for vehicle detection. As shown in Figure 4a, the adaptive ROI is created on top of a fixed trapezoid. A 1-D spatial gradient in the *y* direction is first computed, and the image is then binarized. The adaptive ROI in the figure is related to the width of the vehicle’s vertical edge pair. We define 3×3 and 11×11 convolution masks for erosion and dilation, respectively. Figure 4b shows the results after the morphological operations. The vertical edge pair is then detected by the histogram of vertical projection using several intervals. As illustrated in Figure 4c, using the intervals with different widths on the vertical projection is able to mitigate the influence of noise.

### 4.1. Front Vehicle Detection in Daytime

To detect the front vehicle location, a cumulative density function (CDF) given by

(3) $$\begin{array}{cc} A_{j} & =\left[\Sigma_{i=0}^{j}c_{i}\right]/P_{total} \end{array}$$

is used to search the under-vehicle shadow, where $c_{i}$ is the number of pixels with the intensity value *i*, $P_{total}$ is the total number of pixels in the ROI, and $A_{j}$ is the percentage of pixels with the intensity value less than *j*. Suppose the under-vehicle shadow is in the range of $\left[A_{1},A_{2}\right]$, two thresholds $T_{1}$ and $T_{2}$ are defined by

$$T_{j}=\arg\min_{T}\left\{\sum_{i=0}^{T}\;c_{i}\geq A_{j}\right\}$$

where j=1,2. The threshold for the under-vehicle shadow is then computed by

(4) $$T_{shadow}=T_{1}+\alpha \left(T_{2}-T_{1}\right).$$

Based on our evaluation, the under-vehicle shadow is generally in the range of 0.5% to 15%, and α is set as 0.6.

Given the threshold for under-vehicle shadow detection, the image is binarized, and followed by morphological operations and connected component labeling for noise reduction. The horizontal projection of the shadow is calculated and used to derive the distance of the front vehicle. It is updated continuously and verified with the lane width $W_{lane}$ using

(5) $$\lambda_{1}\cdot W_{lane}<W_{hp}<\lambda_{2}\cdot W_{lane}$$

where $W_{hp}$ is the length of the horizontal projection and $0<\lambda_{1},\lambda_{2}<1$. The front vehicle is then identified based on the symmetry detection using SURF features. We define two 6×3 matrices

$$A=\left[\begin{array}{ccc} a_{02} & a_{01} & a_{00}\\ a_{02} & a_{04} & a_{03}\\ a_{08} & a_{07} & a_{06}\\ a_{11} & a_{10} & a_{09}\\ a_{14} & a_{13} & a_{12}\\ a_{17} & a_{16} & a_{15} \end{array}\right]\;\mathrm{and}\;B=\left[\begin{array}{ccc} b_{00} & b_{01} & b_{02}\\ b_{03} & b_{04} & b_{05}\\ b_{06} & b_{07} & b_{08}\\ b_{09} & b_{10} & b_{11}\\ b_{12} & b_{13} & b_{14}\\ b_{15} & b_{16} & b_{17} \end{array}\right].$$

If there exist SURF features in the 15×15 sub-image block, the values of $a_{i}$ or $b_{i}$ are marked as 1, where i=0,1,⋯,17. Otherwise, their values are denoted by 0. We then check the elements in *A* and *B*. The number of $a_{i}=b_{i}$ is then used to evaluate the possibility of a vehicle.

### 4.2. Front Vehicle Detection in Nighttime

**Figure 5:**
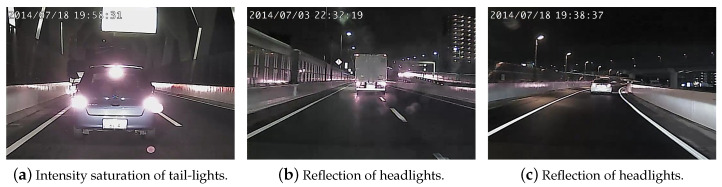
The nighttime images captured by the conventional low dynamic range driving recorder. The intensity saturation commonly appears due to the vehicle tail-lights and the reflection of headlights on the rear bumper.

One major issue of nighttime images acquired by a conventional low dynamic range (LDR) driving recorder is the intensity saturation of vehicle tail-lights, as illustrated in Figure 5a. A similar saturation case is also caused by the reflection of headlights on the rear bumper and tailgate of the front vehicle as shown in Figure 5b,c. To deal with this problem, a nighttime vehicle detection algorithm is proposed. We define an inverted V-shaped ROI and use Equation (1) for image gamma correction. The average intensity of each row in the ROI is calculated, and the brightest area indicates the tail-light location. To suppress the influence of the road surface reflection, the average intensity histogram is generated using several frames. This works well because the front tail-light location usually does not change too much in the image but the ground intensity values tend to have a large variation during the vehicle motion.

**Figure 6:**
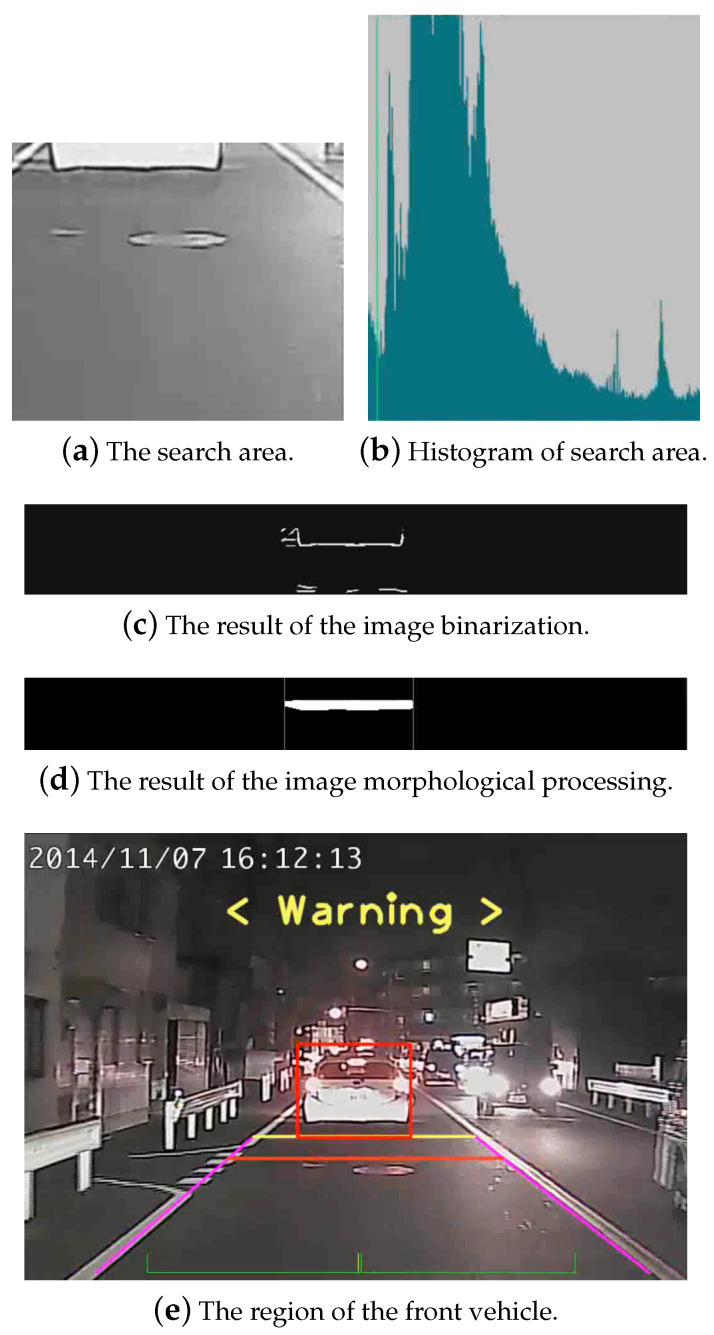
The calculation of the vehicle region using the aspect ratios and intensity histogram in the nighttime images.

Given the vertical location of the brightest area associated with the tail-lights, adaptive thresholding is carried out in the region to obtain a rough vehicle width. To identify the front vehicle in nighttime, we further detect the lower edge of the rear bumper using Equation (3), with $A_{j}$ greater than a threshold. The image is binarized, and followed by morphological processing for noise removal. It is then used to detect the vehicle width by the leftmost and rightmost linked binary pixels. This nighttime vehicle detection process is shown in Figure 6 for a better illustration. Figure 6a,e show the search area and the detected vehicle region, respectively.

## 5. Overtaking Vehicle Detection

**Figure 7:**
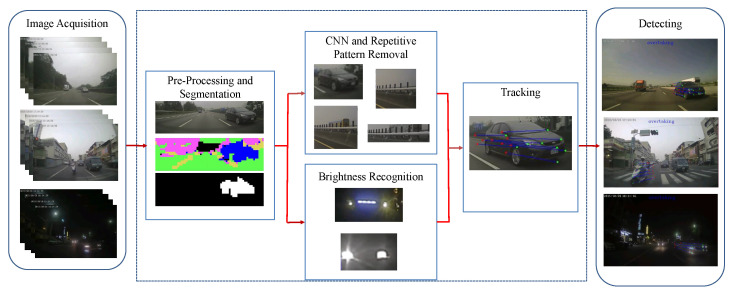
The system flowchart of the proposed overtaking detection technique. It consists of three basic modules: (1) pre-processing and segmentation, (2) convolutional neural network and repetitive pattern removal, and (3) tracking and behavior detection.

The proposed overtaking vehicle detection approach consists of four stages: (1) pre-processing and segmentation, (2) convolutional neural network, (3) repetitive pattern removal, and (4) tracking and behavior detection, as illustrated in Figure 7. A single camera is mounted on the rear windshield for image acquisition.

### 5.1. Pre-Processing and Segmentation

**Figure 8:**
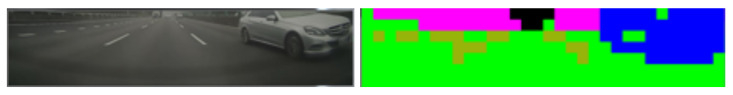
The pavement, lane marking, object approaching from the side, and object further away are shown in green, yellow, blue, and pink, respectively. The black part represents the uncertain region.

The ROI for overtaking vehicle detection is first defined by removing the regions such as sky and buildings, before performing the image segmentation algorithm. To obtain the motion clues in an image, edges and corners are commonly used features for tracking. However, it is difficult to determine the number of features for cluttered scenes, and results in the uncertain computation time. To overcome this problem, we set a fixed number of feature points for each 10×10 sub-image region. The pyramid model of Lucas–Kanade tracker is then used to calculate the optical flow information of the feature points [41]. According to the direction and strength, the results are divided into five categories: (i) road and sky, (ii) lane marking, (iii) overtaking vehicle, (iv) object further away, and (v) uncertain region, as shown in Figure 8.

**Figure 9:**
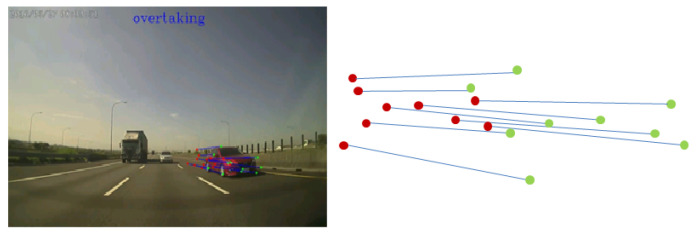
Only the optical flow in the *x*-direction is used to distinguish the overtaking vehicle from the object moving further away.

When moving forward, there is a large amount of optical flow around the vehicle. It is relative small for road and sky, so this characteristic can be used to identify different regions. If the *y*-direction optical flow in the image coordinates is very large and moves toward the vanishing point, the point is classified as lane marking. The points are considered as part of an overtaking vehicle if the *x*-direction optical flow is large and moves away from the vanishing point. On the other hand, it indicates that an object is moving away if the *x*-direction optical flow is large and moves towards the vanishing point. Finally, the situations which do not belong to any of the criteria are assigned to the uncertain region. Here, only the *x*-direction of the optical flow is used to distinguish the overtaking vehicle from the object moving further away, and the *y*-direction optical flow is not taken into account. This is due to the small displacement of motion vector in the *y*-direction, which usually introduces more correspondence mismatching (see Figure 9).

When overtaking vehicles are approaching, the space correlation between the vehicles is used for region filtering. The tracking points of the objects are accumulated to a grayscale image and binarized. A morphological dilation operation is carried out on the tracking points for merging and isolated region removal. The vehicle is then identified using the image segmentation result. One common problem of overtaking scenes is the false correspondence matching between the vehicles and the background. The repetitive patterns generated from constant appearance change will cause incorrect target tracking by the optical flow algorithm. Since the repetitive pattern usually appears in the same image region during a short period of time, it is not possible to use the spatio-temporal correlation of the image pixels to filter out the mismatched features. To deal with this problem, it is necessary to distinguish the repetitive patterns from the features extracted from other meaningful objects such as vehicles, lane markings, etc. The learning-based approaches such as support vector machine (SVM) and neural networks (NN) are commonly adopted in the image classification task. Recently, due to the success of deep neural networks applied to object recognition and classification, large sets of training data and tools are readily available. Thus, a convolutional neural network (CNN) is used for the verification of segmentation results and remove the non-vehicle areas in this paper.

### 5.2. Repetitive Pattern Removal Using Cnn

We adopt CaffeNet as the convolutional neural network architecture in this work. The network is originated from AlexNet [42] and designed to classify 1.2 million high-resolution images in ImageNet LSVRC-2010 contest to 1000 different classes. It consists of five convolutional layers, 60 million parameters and 650,000 neurons. Our dataset is used to fine-tune BVLC CaffeNet model for 6 categories. The output classes contain “front of vehicle”, “rear of vehicle”, “motorcycle”, “repetitive patterns”, “lane markings”, and “background”. The images are segmented by the algorithm described in the previous section, followed by the deep neural network for training and evaluation. The CNN with CaffeNet is used to identify the segmented area and remove the non-vehicle objects.

**Figure 10:**
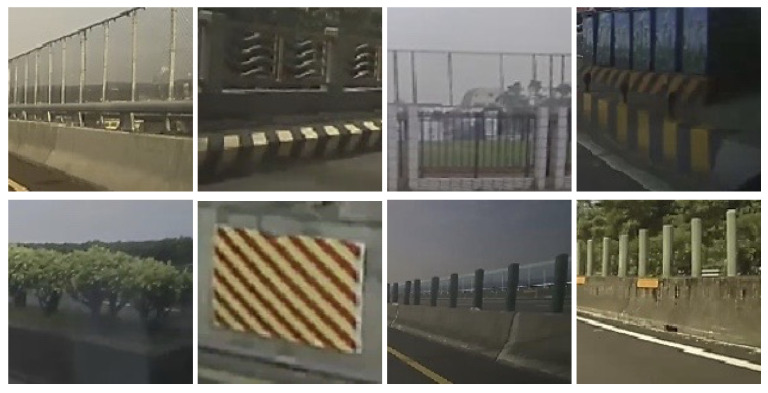
Some common repetitive patterns in the street scenes.

**Figure 11:**
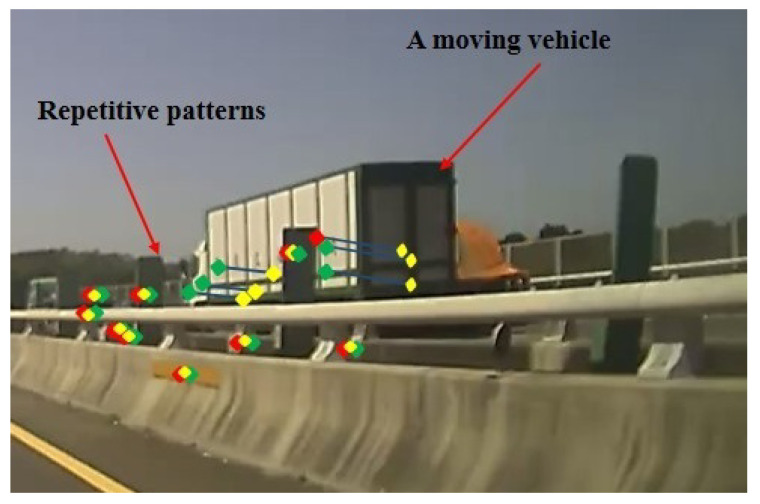
An illustration of messy optical flow when repetitive patterns occur, which result in the forward and backward feature tracking inconsistency.

In some cases, the incorrect segmentation is derived with the vehicle from the opposite lane and recognized by CNN. It is mainly due to the optical flow feature tracking on repetitive patterns as illustrated in Figure 10. There are some common repetitive patterns which can be found in the street scenes. A moving vehicle in the opposite lane might be confused with the repeated pattern due to the separation poles. As shown in Figure 11, the forward and backward feature tracking inconsistency cause the optical flow disorder. To detect whether a segmented region contains repetitive patterns, multiple feature points are adopted for verification. We first detect “good features” to track, denoted by F(t), on the frame at time *t* [43]. The succeeding F(t+1) is then found by referring to the forward optical flow $o_{t}^{+}=\left(u^{+},v^{+}\right)$ from time *t* to t+1. That is,

$$F\left(t+1\right)=F\left(t\right)+o_{t}^{+}\left(F\left(t\right)\right).$$

Similarly, the point $F^{\prime }\left(t\right)$ generated from the backward tracking is related by the backward motion

$$F^{\prime }\left(t\right)=F^{\prime }\left(t+1\right)+o_{t+1}^{-}\left(F^{\prime }\left(t+1\right)\right)$$

where $o_{t+1}^{-}=\left(u^{-},v^{-}\right)$ is the backward optical flow from time t+1 to *t*.

If a feature point F(t) is not part of repetitive patterns and the optical flow is correctly computed, then we have

$$F\left(t\right)-F^{\prime }\left(t\right)=0$$

However, in reality, there are always some errors in the optical flow estimation, i.e.,

$$F\left(t\right)-F^{\prime }\left(t\right)\leq \varepsilon$$

where ε denotes the small error between F(t) and $F^{\prime }\left(t\right)$. Thus, the identified object is classified as a vehicle only if most of the feature points on the target are smaller than ε. Otherwise, it is considered as a repetitive pattern and eliminated from overtaking detection.

**Figure 12:**
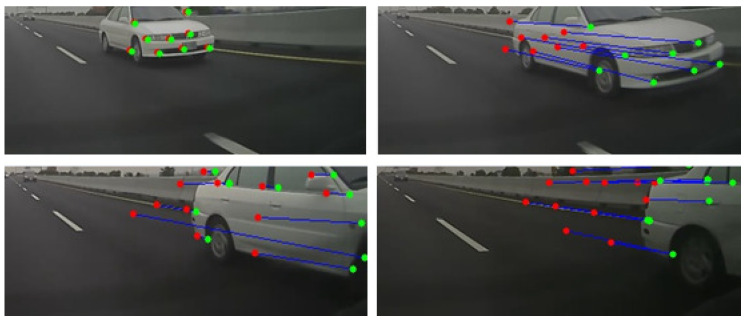
Overtaking tracking diagram. The red point is the initial position, and the green point is the current position. The blue line is the corresponding line.

After region segmentation and repetitive pattern removal, the object tracking is carried out until the object is disappeared from the image or no overtaking occurs. We adopt Lucas–Kanade optical flow for continuously tracking to overcome the shape, size, and scale of the object changes as it approaches the camera. A tracking example is shown in Figure 12. It is expected that CNN is able to identify and remove repetitive patterns, and provide the correct overtaking detection. However, due to the low image resolution, the region segmentation is not always good enough for identification. To reduce the false detection, we further assess whether the tracking direction continuously stays away from the vanishing point for a period of time.

For nighttime overtaking detection, the vehicle appearance cannot be clearly obtained due to the low illumination environment. It is also easily disturbed by other background lights if the vehicle headlights are used for target detection. To identify the overtaking vehicles, we present a method which uses motion cues combined with the brightness information. The approaching headlight areas are segmented using the algorithm described in Section 5.1. We binarize the images and track the brightness region, followed by the vehicle motion analysis presented previously. It is considered as the headlight of an overtaking vehicle if continuously moving away from the vanishing point.

## 6. Implementation and Experiments

The proposed technique adopts front and rear cameras to capture the video sequence and is used for lane marking, front vehicle, and overtaking vehicle detection.

### 6.1. Camera Setting and System Calibration

To make the system installation flexible and easy to use, we develop an auto-calibration mechanism for camera parameter estimation. Two commonly adopted camera settings, looking upward or downward, are considered, and the error introduced by imprecise system setup is analyzed. Assume a camera with the focal length *f* is installed on a vehicle at the height β. We first consider the case where the camera faces downward with an angle $\theta_{v}$ with respect to the ground plane. Then we have

(6) $$\theta_{v}=\tan^{-1}\frac{2h_{v}-h}{2f}$$

where *h* is the image height and $h_{v}$ is the vanish point location in the image.

Assuming an object with the physical size λ is captured by a camera with the dimension $\lambda_{o}$ in the image, then

(7) $$\theta_{\lambda }=\tan^{-1}\frac{2h_{\lambda }-h}{2f}.$$

If we further assume that the object is located in front of the camera at the distance γ, then we have

(8) $$\beta =\lambda -\gamma \tan(\theta_{\lambda }-\theta_{v})$$

Since the vanishing point $h_{v}$ can be obtained using the parallel lane markings on the ground plane, the camera tilt angle $\theta_{v}$ can be derived from Equation (6). If a calibration object is given with known size and distance, the camera’s position can be computed by Equations (7) and (8). Finally, the one-to-one correspondence between the image plane and the ground can be established, and the distance can be derived directly from the acquired image.

Similarly, for the case with the camera looking upward, the parameters $\theta_{v}$, $\theta_{\lambda }$, and β are calculated by

$$\theta_{v}=\tan^{-1}\frac{h-2h_{v}}{2f},\;\theta_{\lambda }=\tan^{-1}\frac{2h_{\lambda }-h}{2f}$$

and

$$\beta =\lambda -\gamma \tan(\theta_{\lambda }+\theta_{v})$$

respectively. The camera’s installation position and orientation can then be obtained accordingly.

### 6.2. Experiments and Evaluation

**Figure 13:**
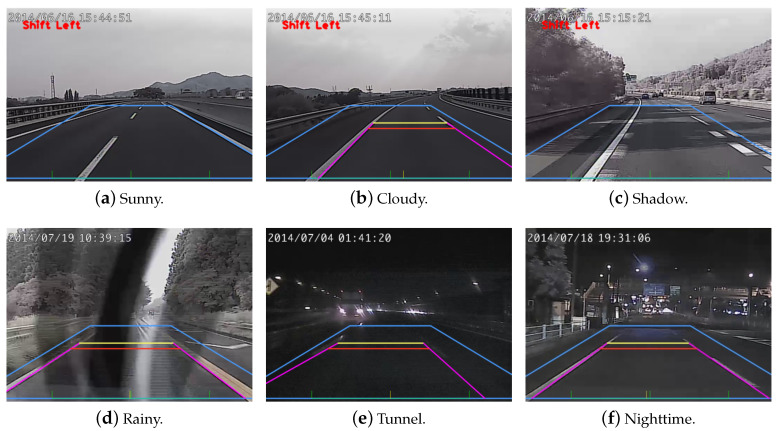
The results of lane change detection. The images contain a variety of illumination conditions such as sunny, cloudy, and at night.

**Figure 14:**
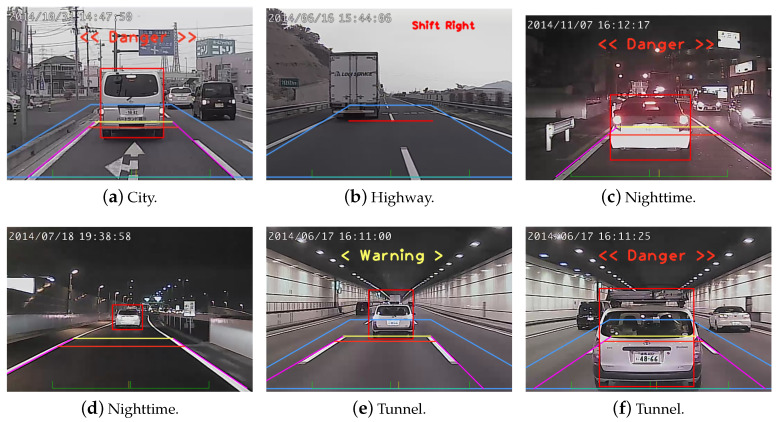
The results of forward collision warning. The images contain city, highway, nighttime, and tunnel traffic scenes.

The proposed methods for lane change detection, forward collision warning, and overtaking vehicle identification have been tested using car digital video recorders (DVR) developed by Create Electronic Optical. Two DVRs are mounted behind the front and rear windshield to capture the image sequences for performance evaluation. The original image size and the sub-image region for processing are 1200×800 and 600×200, respectively. Our processing platform is a PC with Ubuntu 16.04 operating system, 4.0 GHz Intel i7 CPU, and Nvidia GTX 1080 GPU. Some results of lane change detection and forward collision warning are shown in Figure 13 and Figure 14. The test video sequences cover a variety of illumination conditions such as sunny, cloudy or rainy weather, in a tunnel, or during the night time. Two horizontal lines, yellow and red, indicate the distances of 30 m (warning) and 50 m (danger), respectively. They represent an industrial standard used to generate the warning signals.

The performance of lane change detection and forward collision warning is evaluated based on the detection accuracy. Since it is usually not necessary and mostly infeasible to evaluate the system accuracy for all possible situations, some criteria are adopted as follows:

1. The width of the lanes is 3∼4 m.
2. The vehicle velocity is over 60 km/h.
3. The curvature of the lanes is over 250 m in radius.
4. The visible range of the camera is over 120 m.
5. The toll booth areas are not considered.

In the above, the lane width is based on the transportation infrastructure. The consideration of vehicle velocity, road curvature, toll booth area, and camera visible range are from the application specifications. There are a total 2738 images used for lane change detection in our experiment. The precision and recall of the proposed technique and the previous methods are tabulated in Table 1. For forward collision warning, there are 3464 images used in this work. Table 2 shows the precision and recall of previous techniques and our proposed method. It indicates that, compared to others, our results provide better precision but not the recall rate.

**Figure 15:**
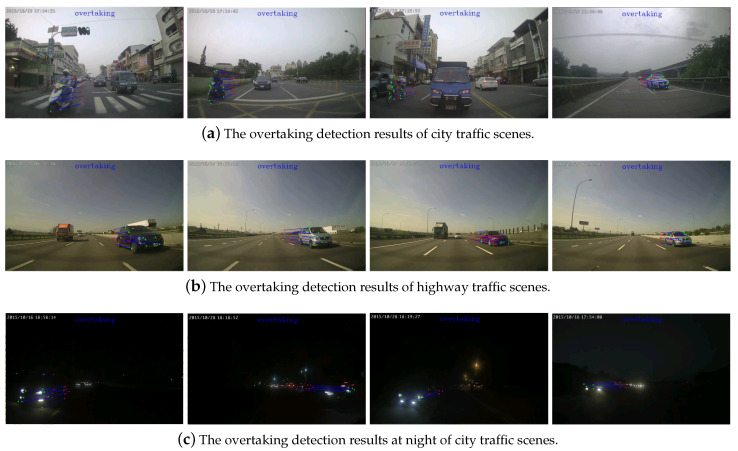
The overtaking detection results for the city, highway, and night scenery.

For overtaking detection, there are totally 7587 images collected in our training data with 6 categories. The images are manually segmented and labeled with 808 lane markings, 470 motorcycles, 2405 repetitive patterns, 1301 rear of vehicle, 1385 front of vehicle, and 1218 background. Similarly, the validation data of 2770 images are collected from 80-minute video sequences and marked manually. The recognition results for all categories are tabulated in Table 3. For performance evaluation, 50 image sequences (20 highway, 15 city, 15 night) are collected with each about 2 min long. The overtaking detection is successful if the vehicle is detected before disappeared from the image sequence. It is considered as failed if the approaching vehicle is not identified. The overtaking evaluation is tabulated in Table 4 and some result images are shown in Figure 15 for the city traffic, highway, and at night, respectively. Figure 15a shows the city traffic scenes with overtaking motorcycles and vehicles detected. It is noted that the vehicles behind but without overtaking or driving in the opposite direction are not identified. Figure 15b shows the highway traffic scenes with correct overtaking detection even with the repetitive pattern of the separation poles appeared in the images. The overtaking vehicles in the night scenes are identified by the headlight features as shown in Figure 15c. In our vehicle recognition module, it does not matter if the overtaking is identified or not since a subsequent tracking stage will be performed. However, a false alarm will occur if the vehicle makes turns with another behind. The previous works on overtaking vehicle identification are fairly limited. In [53], the results report the overtaking detection with 98 true positives, 5 false positives, 3 false negatives, at the precision and recall rates of 95.1% and 97.0%, respectively. The work in [54] shows the precision and recall rates of 96.3% and 86.9%, respectively. In the proposed method, the precision rates for ’city traffic’, ’highway’, and ’night’ are 88.7%, 98.8%, and 90.2%, respectively. The recall rates for these three scenarios are 100%, 100%, and 88.1%, respectively.

## 7. Conclusions and Future Work

In this paper, we propose a vision-based driver assistance system for highway, urban, and city environments. The efficient and robust techniques for lane change detection, forward collision warning, and overtaking vehicle identification are presented. We adopt two monocular car digital video recorders to capture the front and rear views of the traffic scenes. In our proposed approach, the front vehicles are identified by a new CDF-based symmetry detection technique. For overtaking detection, the motion cue obtained from optical flow is combined with convolutional neural networks for vehicle identification with repetitive patterns removal. In the system setup, the on-board camera projection geometry is formulated and the camera calibration is performed for single view depth measurement. The experiments and evaluation carried out on various real traffic scenarios have demonstrated the effectiveness of the proposed techniques.

In the future work, there are a few important research directions for practical uses in real-world application scenarios. First, the lane change detection can be improved with road surface segmentation. Since lane markings are not always clearly available, the drivable area should be considered for lane change assistance. Second, stereo vision can be adopted for more accurate depth estimation of the front vehicles. It will provide more reliable information for integration with other tasks such as cruise control. Third, the current overtaking detection technique does not perform very well when the vehicle makes turns. The ego-motion estimation can be carried out to eliminate the incorrect feature matching for overtaking vehicle identification. Finally, the proposed techniques aim to be implemented on an embedded platform and evaluated for the real-time capability.

## Figures and Tables

**Table 1:** The comparison of precision and recall of lane change detection for different algorithms. T.P.: true positive, F.P.: false positive, F.N.: false negative. Some previous works do not provide the numbers of true positives, false positives, false negatives, and recall rates.

Method	T.P.	F.P.	F.N.	Precision	Recall
Woo et al. [44]	–	–	–	96.3%	100%
Mandalia et al. [45]	–	–	–	80.0%	80.5%
Schlechtriemen et al. [46]	–	–	–	93.6%	99.3%
Aly et al. [47]	1955	235	–	96.4%	–
Ye et al. [48]	1998	48	–	98.5%	–
Proposed method	2423	53	80	97.9%	96.8%

**Table 2:** The comparison of precision and recall of front vehicle detection for different algorithms. T.P.: true positive, F.P.: false positive, F.N.: false negative. Some previous works do not provide the numbers of true positives, false positives, and false negatives.

Method	T.P.	F.P.	F.N.	Precision	Recall
Qu et al. [49]	–	–	–	90.3%	80.5%
Chen et al. [50]	–	–	–	90.8%	78.8%
Li et al. [51]	–	–	–	90.4%	79.2%
Yang et al. [52]	–	–	–	91.9%	83.6%
Proposed method	1510	0	911	100%	62.4%

**Table 3:** The recognition results for each category and the recall. GT: ground-truth, BG: background, LM: lane marking, MC: motorcycle, RP: repetitive pattern, FV: front of vehicle, RV: rear of vehicle.

	BG	LM	MC	RP	FV	RV
GT	375	252	545	408	480	710
BG	322	1	2	0	1	11
LM	2	249	0	2	0	0
MC	0	0	524	1	0	0
RP	25	2	0	405	0	3
FV	13	0	16	0	470	34
RV	13	0	3	0	9	662
Precision	85.9%	98.8%	96.1%	99.3%	97.9%	93.2%
Recall	85.8%	98.8%	96.1%	99.2%	97.9%	93.2%

**Table 4:** The experimental results and performance evaluation.

Scene	True Overtakes	Detected	Missed	False
City traffic	102	102	0	13
Highway	79	79	0	1
At night road	42	37	5	4
